# Photocatalytic Oxidation of Diethyl Sulfide Vapor over TiO_2_-Based Composite Photocatalysts

**DOI:** 10.3390/molecules191221424

**Published:** 2014-12-19

**Authors:** Dmitry Selishchev, Denis Kozlov

**Affiliations:** 1 Boreskov Institute of Catalysis, pr. Ak. Lavrentieva 5, Novosibirsk 630090, Russia; 2 Novosibirsk State University, st. Pirogova 2, Novosibirsk 630090, Russia; 3 Research and Educational Centre for Energoefficient Catalysis (NSU), st. Pirogova 2, Novosibirsk 630090, Russia

**Keywords:** photocatalysis, TiO_2_, activated carbon, silica, composite photocatalyst, diethyl sulfide

## Abstract

Composite TiO_2_/activated carbon (TiO_2_/AC) and TiO_2_/SiO_2_ photocatalysts with TiO_2_ contents in the 10 to 80 wt. % range were synthesized by the TiOSO_4_ thermal hydrolysis method and characterized by AES, BET, X-ray diffraction and FT-IR ATR methods. All TiO_2_ samples were in the anatase form, with a primary crystallite size of about 11 nm. The photocatalytic activities of the TiO_2_/AC and TiO_2_/SiO_2_ samples were tested in the gas-phase photocatalytic oxidation (PCO) reaction of diethyl sulfide (DES) vapor in a static reactor by the FT-IR *in situ* method. Acetaldehyde, formic acid, ethylene and SO_2_ were registered as the intermediate products which finally were completely oxidized to the final oxidation products – H_2_O, CO_2_, CO and SO_4_^2−^ ions. The influence of the support on the kinetics of DES PCO and on the TiO_2_/AC and TiO_2_/SiO_2_ samples’ stability during three long-term DES PCO cycles was investigated. The highest PCO rate was observed for TiO_2_/SiO_2_ photocatalysts. To evaluate the activity of photocatalysts the turnover frequency values (TOF) were calculated for three photocatalysts (TiO_2_, TiO_2_/AC and TiO_2_/SiO_2_) for the same amount of mineralized DES. It was demonstrated that the TOF value for composite TiO_2_/SiO_2_ photocatalysts was 3.5 times higher than for pure TiO_2_.

## 1. Introduction

Volatile organic compounds containing N, S, P or Cl heteroatoms are often highly toxic and very dangerous for human health [1,2,3], and some of them could be used as chemical warfare agents (CWA) [4]. One of the best known CWAs is bis(2-chloroethyl) sulfide or mustard gas (HD). This species is a highly toxic vesicant which causes destruction of cell membranes and nucleic acids. It binds with nucleophilic groups like sulphur atoms in the SH-groups of proteins and nitrogen atoms in the nitrogen bases of DNA [5]. The relative toxicity (LD_50_) for HD inhalation is about 1.5 mg·min/L and this value is the highest among vesicants [6]. In this way the development of effective methods for HD neutralization is an important task to ensure human safety.

The main chemical methods of HD detoxification include nucleophilic substitution or oxidation, which result in the cleavage of C-S or C-Cl bonds and partial or even complete oxidation of the target molecule [7]. HD can be destroyed by photolysis or oxidized by photogenerated ozone under UV light irradiation [8]. At the same time the method of photocatalytic oxidation using TiO_2_ as the photocatalyst is regarded as one of the promising methods of CWA disposal due to the high oxidative ability of TiO_2_ under UV irradiation [9]. PCO makes it possible to destroy dangerous compounds completely with the formation of CO_2_, H_2_O, ${\mathrm{NO}}_{3}^{-}$, ${\mathrm{SO}}_{4}^{2-}$, ${\mathrm{PO}}_{4}^{3-}$ and Cl^−^ as final products [10].

In the view of the high toxicity of HD, researchers usually use for laboratory investigations simulants such as 2-chloroethyl ethyl sulfide [11], 2-chloroethyl methyl sulfide [12], 2-phenethyl 2-chloroethyl sulfide [13], diethyl sulfide (DES) [14] and dimethyl sulfide [15]. These simulants are safer due to their lower toxicity, but at the same time they simulate well the chemical behavior of HD.

In the current work we focused on the investigation of DES vapor PCO. In our previous works we demonstrated that DES can be easily decontaminated under the UV irradiation using TiO_2_ as the photocatalyst with the formation of CO_2_, H_2_O and surface sulfate and carbonate species as the final PCO products [14,16,17,18]. Acetaldehyde, ethanol, ethylene, SO_2_ and other trace products were detected as the gas-phase intermediates, while polysulfides, diethyl sulfone, diethyl sulfoxide were detected as the surface intermediates. All intermediates were completely oxidized to the final products after long-term irradiation [14,16]. Analysis of intermediates and products allowed the authors to propose the main routes of the DES PCO, which include C-S bond cleavage, and oxidation of sulfur and carbon atoms.

To enhance the rate of air purification from DES vapor in a closed chamber a TiO_2_ aerosol generated by a sonic method could be applied [19]. Aerosol spraying led to the fast adsorption of DES vapor and its further photocatalytic oxidation under UV irradiation.

The main problem during the long-term DES oxidation is the deactivation of the TiO_2_ photocatalyst. An increase of the time required for the complete mineralization of DES was clearly seen from the kinetic curves of CO_2_ accumulation during several oxidation cycles in a batch reactor [14]. FT-IR analysis demonstrated that the accumulation of non-volatile organic intermediates like polysulfides, diethyl sulfone, diethyl sulfoxide and sulfate species on the surface of photocatalyst are responsible for its deactivation during the long-term experiments. The positive influence of using the composite TiO_2_/adsorbent photocatalysts was also discussed in our previously published paper devoted to the computer simulation of the kinetics of photocatalytic reactions [20] where we demonstrated an increase of the rate of substrate removal for TiO_2_/adsorbent photocatalysts.

In recent years composite photocatalysts in which TiO_2_ is deposited onto the surface of a porous support like activated carbon (AC), silica or zeolite were actively investigated in the PCO processes of various pollutants, both in the gas and liquid phases [21,22,23,24,25,26]. In addition to the increase of the adsorption capacity in some cases the increase of the PCO rate and the decrease of deactivation degree were observed for such composite photocatalysts [27,28,29].

Concerning mustard gas, several research groups have studied PCO of HD simulants using composite photocatalysts. Cr-modified TiO_2_-loaded MCM-41 silica photocatalyst was studied in the oxidation of DES vapor in a batch reactor under UV irradiation [30]. It was demonstrated that the TiO_2_ deposition on the Cr-MCM-41 support increases the DES removal rate, but decreases the CO_2_ formation rate if compares with the commercial Hombifine N TiO_2_ (Sachtleben Chemie GmbH, Duisburg, Germany).

Panayotov and co-workers investigated the PCO of 2-chloroethyl ethyl sulfide (CEES) and DES on a mixed oxide TiO_2_-SiO_2_ photocatalyst [31,32,33]. They revealed that the CEES adsorbs on the surface of the composite photocatalyst through both the chlorine and sulfur atoms by bonding to isolated OH groups. The authors also demonstrated that the presence of the Cl atom in the CEES molecule does not significantly influence the PCO rate if compared with the DES molecule. Partially or fully oxidized products were observed during the photooxidation of both tested molecules over the composite photocatalyst. Partially oxidized products have been demonstrated to block OH groups on the surface of photocatalysts and to prevent further adsorption of target molecules and to thus reduce the rate of photooxidation. Unfortunately, no comparison between pure TiO_2_ and composite TiO_2_-SiO_2_ photocatalyst was done. The main drawback of the previous works is the absence of systematic investigations of the behavior of composite photocatalysts during the long-term PCO of HD simulants.

In spite of the fact that AC is the most frequently used TiO_2_ support in composite photocatalysts, the SiO_2_ material is also promising due to its higher hydrophilicity, transparency and quantity of OH-groups. In this connection, the main objective of the current study was to investigate the PCO of DES in the gas-phase over composite photocatalysts in which TiO_2_ was deposited onto AC or SiO_2_ surfaces. We investigated the effect of the porous support on the kinetics of DES PCO and on the composite photocatalyst activity in multiple long-term experiments. Finally, a comparison between pure TiO_2_ and TiO_2_/adsorbent photocatalysts was made.

## 2. Results and Discussion

### 2.1. Characterization of the Synthesized Photocatalysts

Synthesis of TiO_2_, TiO_2_/AC and TiO_2_/SiO_2_ samples was performed by the TiOSO_4_ thermal hydrolysis method. This method has some advantages in comparison with the popular sol-gel method which utilizes titanium alkoxides because titanyl sulfate is a cheaper precursor. Synthesized TiO_2_ samples were of anatase crystal structure with a high surface area and good crystallinity. As a result the TiO_2_ photocatalyst synthesized by this procedure usually have high photocatalytic activity in the oxidation of volatile organic compounds [34].

In our previous work TiO_2_/AC samples with TiO_2_ contents higher than 60 wt. % demonstrated high photocatalytic activity [22]. Therefore in this work we prepared TiO_2_/AC samples with estimated TiO_2_ contents equal to 65, 70 and 80 wt. %. The TiO_2_/SiO_2_ photocatalysts have high photoactivity even at a relatively low TiO_2_ content, so we prepared several TiO_2_/SiO_2_ samples with estimated TiO_2_ contents in the range from 10 to 80 wt. %. Varying the TiO_2_ content in the series of TiO_2_/adsorbent samples allows us to choose a photocatalyst with high adsorption capacity and at the same time with high photocatalytic activity for further investigation of DES PCO. The results of AES and BET analysis are presented in the Table 1.

**Table 1:** TiO_2_ content and textural properties of the samples.

Series	Sample *	TiO_2_ Content, wt. %	Surface Area, m^2^/g	Pore Volume, cm^3^/g
Supports	AC	--	825	0.54
	SiO_2_	--	442	0.78
TiO_2_	TiO_2_	100	208	0.15
TiO_2_/AC	80-TC	77.5	299	0.22
	70-TC	68.5	367	0.27
	65-TC	62.5	401	0.26
TiO_2_/SiO_2_	80-TS	76.5	237	0.27
	60-TS	57.5	270	0.37
	40-TS	39.7	298	0.48
	20-TS	22.2	351	0.58
	10-TS	12.3	396	0.68

* Number in the sample label indicates the estimated TiO_2_ content, wt. %.

**Figure 1:**
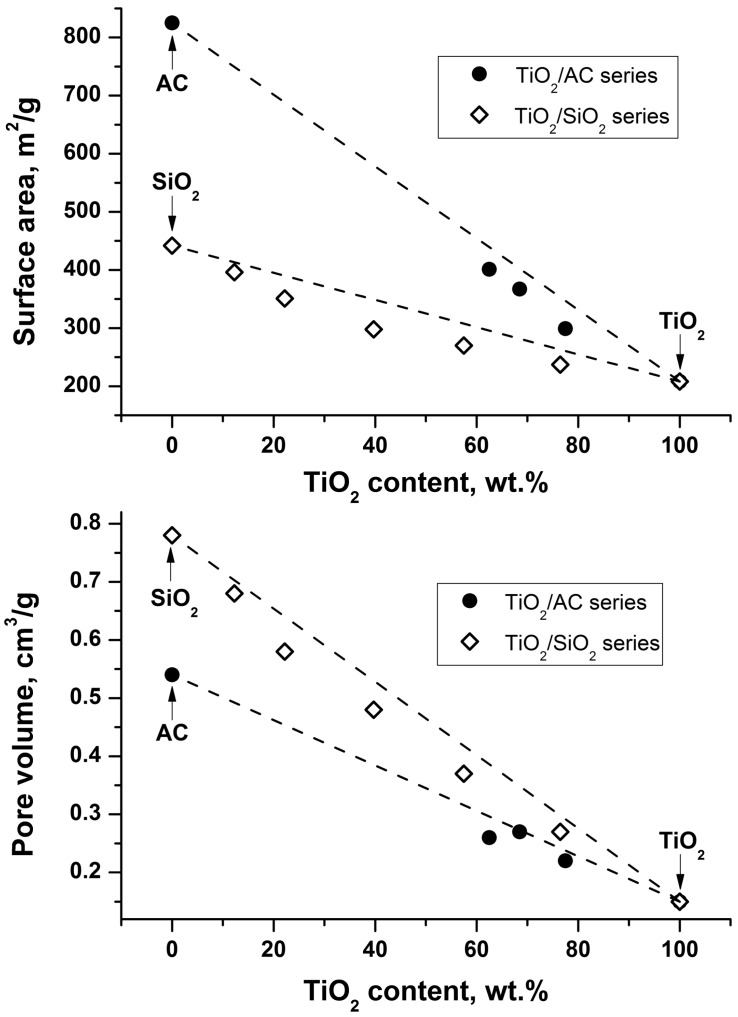
Dependences of the specific surface area and pore volume on TiO_2_ content for the TiO_2_/AC and TiO_2_/SiO_2_ composite photocatalysts.

It follows from the Table 1 that the synthesized TiO_2_ sample has a high surface area (208 m^2^/g) and pore volume (0.15 cm^3^/g). The corresponding values for composite TiO_2_/AC and TiO_2_/SiO_2_ photocatalysts are higher because of the higher porosity of AC and SiO_2_. Moreover, the lower the TiO_2_ content is the higher specific surface area and pore volume of the composite photocatalyst are (Figure 1). Figure 1 demonstrates that the specific surface area and pore volume of the composite photocatalyst are slightly lower than the algebraic sum of the corresponding values of TiO_2_ and adsorbent (AC or SiO_2_). This means that a partial blocking of the support surface by TiO_2_ nanoparticles occurs.

**Figure 2:**
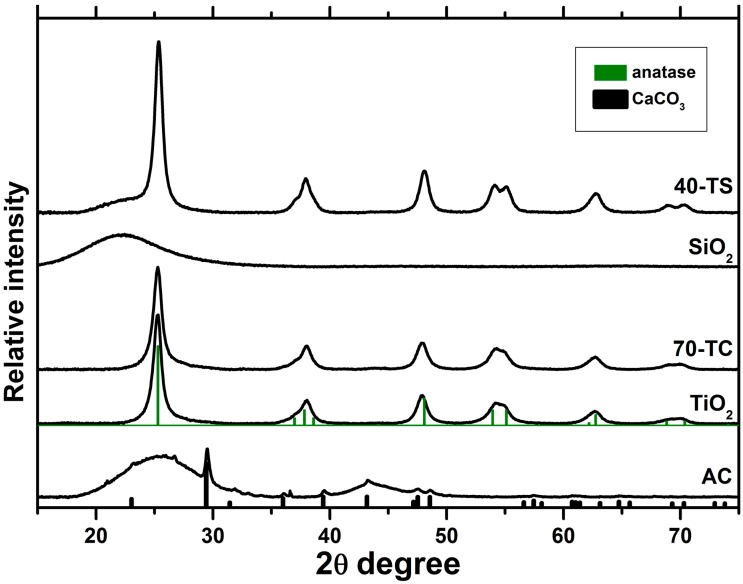
XRD patterns for the pure TiO_2_, AC, SiO_2_ and for the composite 70-TC and 40-TS photocatalysts.

XRD patterns for pure TiO_2_ and composite photocatalysts are presented in Figure 2. It can be seen that the XRD patterns of TiO_2_/AC catalyst only have anatase peaks while broad activated carbon peaks are not detected due to the low AC content. Also a small amount of CaCO_3_ admixture is observed in the AC sample.

In the TiO_2_/SiO_2_ samples, in addition to anatase peaks, there appears a broad silica peak at the 2θ value equal to 20–30° indicating its amorphous structure. This silica peak overlaps with the 2θ = 25.3° peak of anatase. By and large the anatase peaks are similar for pure TiO_2_ and composite TiO_2_/AC and TiO_2_/SiO_2_ samples which indicates that TiO_2_ crystallites have the same size in all cases because the value of the coherent-scattering domains size is about 11 nm for all samples.

**Figure 3:**
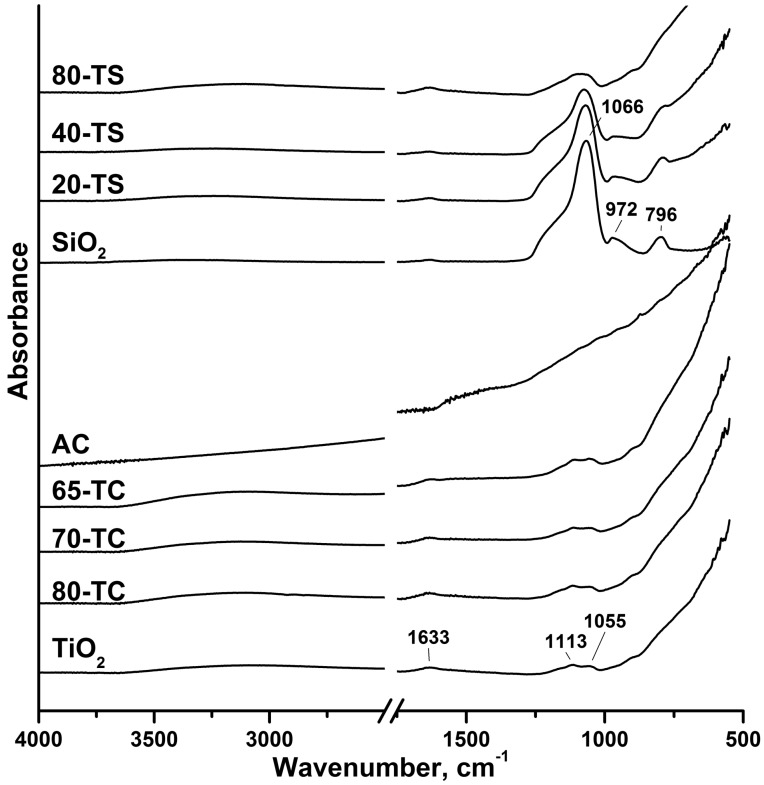
ATR FT-IR spectra for pure TiO_2_, AC, SiO_2_ and for the composite photocatalysts.

Figure 3 shows the IR spectra of all synthesized photocatalysts, AC and SiO_2_ supports measured by the FT-IR ATR technique. Since all measurements were carried out under ambient conditions the water δ_s_(H_2_O) absorption band at 1633 cm^−1^ was recorded in all samples except for AC powder. A broad absorption band in the 2800–3750 cm^−1^ range corresponds to the stretching vibration of the surface OH-groups and physically adsorbed H_2_O molecules.

The 1055 and 1113 cm^−1^ absorption bands in the spectrum of the pure TiO_2_ sample correspond to the vibrations in sulfate complexes [35]. The presence of sulfur was additionally confirmed by the atomic emission spectroscopy (AES) results, which revealed about 1.3 wt. % of S. This means that bonded sulfate complexes remain on the catalyst surface even after thorough washing. The presence of sulfate groups on the photocatalyst surface was also observed for the TiO_2_/AC sample. For the TiO_2_/SiO_2_ catalysts identification of surface sulfate group was difficult because their signals overlapped with the stretching vibration bands of Si-O-Si and Si-O-H bonds near the 1000 cm^−1^ region.

### 2.2. Kinetic Experiments

The main purpose of our work was to study the PCO of diethyl sulfide with the composite photocatalysts and to investigate their stability in long-term experiments. In this connection in the beginning we optimized the quantity of the photocatalyst. Then we chose the photocatalyst with adsorptivity and photocatalytic activity in good proportions and finally we investigated its stability in the DES PCO.

#### 2.2.1. Effect of the Sample Quantity on the Photocatalytic Activity

The photocatalytic activities of pure TiO_2_ and composite samples were measured in a continuous flow reactor in the reaction of acetone vapor PCO. The CO_2_ formation rate was used as a measure of the photocatalytic activity. Photocatalysts were uniformly deposited onto a 3 × 3 cm glass support and then installed into the continuous flow reactor (see Experimental Section). The quantity of photocatalyst deposited was measured in mg/cm^2^ units. For the quantity optimization experiments several glass supports with different quantities were prepared for all photocatalysts. For the pure TiO_2_ the quantities were 0.25, 0.5, 1, 2 and 3 mg/cm^2^. For the composite samples their quantities were adjusted in such a way that the quantities of contained TiO_2_ were in the 0.2–3 mg/cm^2^ range. For example the 40-TS sample contains 39.7 wt. % of TiO_2_ (Table 1) and to achieve the 0.25 mg/cm^2^ value a quantity of 0.25/0.397 = 0.63 mg/cm^2^ of 40-TS sample was deposited onto the glass support.

**Figure 4:**
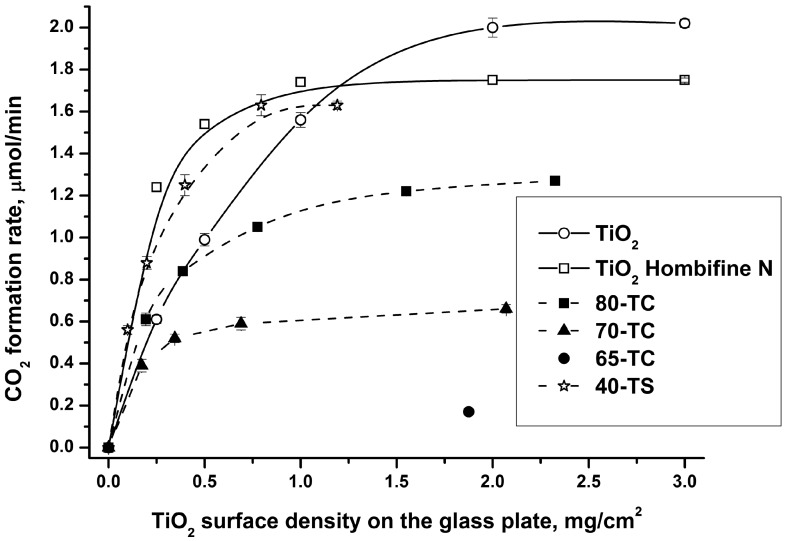
Influence of the quantity of photocatalyst on its photocatalytic activity.

Figure 4 demonstrates the dependencies of the steady-state rate of CO_2_ formation during acetone oxidation over TiO_2_, TiO_2_/AC and TiO_2_/SiO_2_ samples on the quantity of contained TiO_2_. The higher is the contained TiO_2_ on the glass support, the thicker the photocatalyst layer is.

It could be seen that in all cases the PCO rate achieves the maximum value. It corresponds to the situation when the incident light is completely absorbed by the photocatalyst and any further increase in the quantity of photocatalyst leads to the formation of the bottom unirradiated photocatalyst layers which do not work.

The quantity of photocatalyst which corresponds to the maximum PCO rate depends on the TiO_2_ content and its dispersion. For example for the commercial Hombifine N TiO_2_ (Sachtleben Chemie GmbH, 100% anatase, S_BET_ = 350 m^2^/g) the maximum rate quantity is about 1 mg/cm^2^, whereas for the synthesized TiO_2_ sample it is about 2 mg/cm^2^.

It should be noted that at low sample quantity the 40-TS sample is more active than pure TiO_2_. As it follows from the Table 1 both photocatalysts have the same size of TiO_2_ crystallites—11 nm—therefore the difference of activities could be explained by a higher dispersion of the TiO_2_ particles deposited onto the silica in the 40-TS sample than in the pure TiO_2_.

On the other hand activities of the TiO_2_/AC samples are lower than for pure TiO_2_ because unlike silica, AC absorbs UV irradiation. Due to low photocatalytic activity of TiO_2_/AC photocatalysts we used samples 80-TC and 70-TC with high TiO_2_ content. Two conclusions could be reached from the above discussion:

(1) Comparison of the photocatalysts’ activity should be done using high quantities when the thickness of the photocatalyst layer is sufficient for complete light absorption (e.g., 2–3 mg/cm^2^). We used this approach when choosing a photocatalyst with good adsorptivity and photocatalytic activity (see Section 2.2.2);
(2) Studies of long-term photocatalyst use should be done using a relatively low TiO_2_ quantity (e.g., 0.5 mg/cm^2^) because in this case we can assume that the entire photocatalyst surface is irradiated and is involved in the reaction process. This is the reason why we investigated the diethyl sulfide oxidation with a 0.5 mg/cm^2^ quantity of TiO_2_.

#### 2.2.2. Effect of TiO_2_ Content on the Photocatalytic Activity of the TiO_2_/SiO_2_ Catalyst

**Figure 5:**
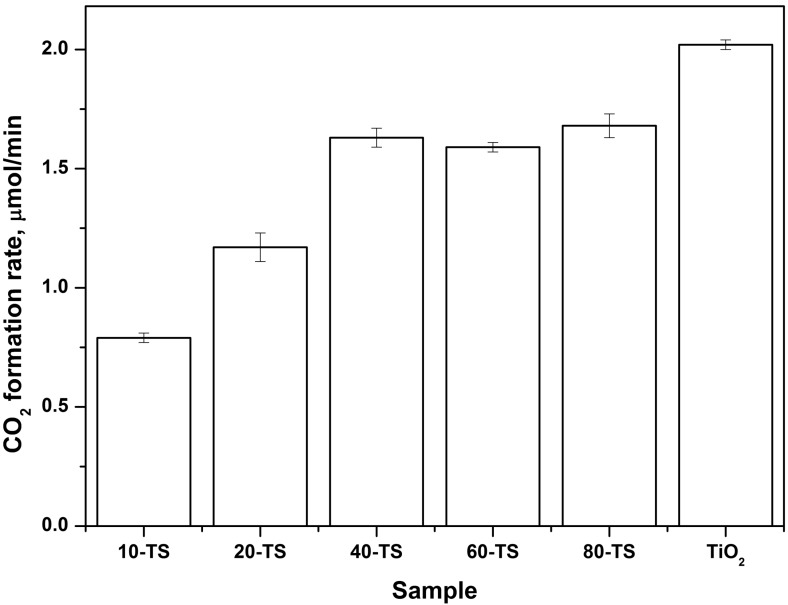
Dependence of the CO_2_ formation rate during acetone PCO on the TiO_2_ content for TiO_2_/SiO_2_ series.

Figure 5 demonstrates dependencies of the steady-state rate of CO_2_ formation during acetone oxidation over the TiO_2_/SiO_2_ photocatalysts. The quantity of catalysts on the glass supports in these experiments was 3 mg/cm^2^ in order to compare the highest possible photocatalytic activity of the samples.

All TiO_2_/SiO_2_ samples demonstrate high activity, even at a low TiO_2_ content, because silica does not absorb UV light. The CO_2_ formation rate for the 10-TS sample which contains 12 wt. % of TiO_2_ was 0.79 μmol/min and it was only 2.5 times lower than for a pure TiO_2_ sample.

The oxidation rate increases with the increase of TiO_2_ content and achieves almost the highest value for the 40-TS sample with 40 wt. % TiO_2_ content. The 60-TS and 80-TS samples have slightly higher activity and it means that at 40 wt. % TiO_2_ content SiO_2_ particles are already completely covered with the TiO_2_ particles. Therefore the following DES PCO experiments were conducted with the 40-TS sample which demonstrated high adsorption capacity due to its high content of porous support and at the same time high photocatalytic activity.

#### 2.2.3. Kinetics of the DES PCO in a Static Reactor

The main objective of the experiments in the static reactor was to compare the kinetics of DES oxidation over pure TiO_2_ and composite TiO_2_/AC and TiO_2_/SiO_2_ photocatalysts and to compare the photocatalysts’ deactivation during three consecutive DES PCO cycles.

Composite 80-TC, 70-TC and 40-TS samples as well as pure TiO_2_ photocatalyst were chosen for these investigations. Sample quantities were correspondingly adjusted to 0.65, 0.73, 1.3, and 0.5 mg/cm^2^ for 80-TC, 70-TC, 40-TS and pure TiO_2_, so that the net TiO_2_ quantity was equal to 0.5 mg/cm^2^ in all cases. The same amount of active component (*i.e.*, TiO_2_) placed in the reactor allowed us to carry out a valid comparison of photocatalyst deactivation for the samples with different TiO_2_ content and to estimate the effect of the support.

Н_2_O, CO_2_ and СO were detected as final gaseous oxidation products. The concentration of CO did not exceed the 55 ppm level and it was which much lower than the final CO_2_ concentration which was equal to about 1400 ppm. The final surface products of DES PCO were sulfate complexes. The accumulation of sulfates on the photocatalysts surface was confirmed by FT-IR analysis and it was the reason of irreversible photocatalysts deactivation.

Acetaldehyde (CH_3_CHO), formic acid (HCOOH), ethylene (C_2_H_4_) and SO_2_ were detected in the gas phase as intermediates of DES PCO. All intermediates were completely oxidized to final products during the long-term irradiation. Noticeable concentrations were detected only for acetaldehyde and formic acid, therefore their kinetic curves were discussed along with DES removal and CO_2_ accumulation kinetic curves.

SO_2_ was detected in the gas phase only during the first PCO cycle and its concentration did not exceed the 30-50 ppm level. Quantitative analysis of ethylene was not performed due to its low concentration.

**Figure 6:**
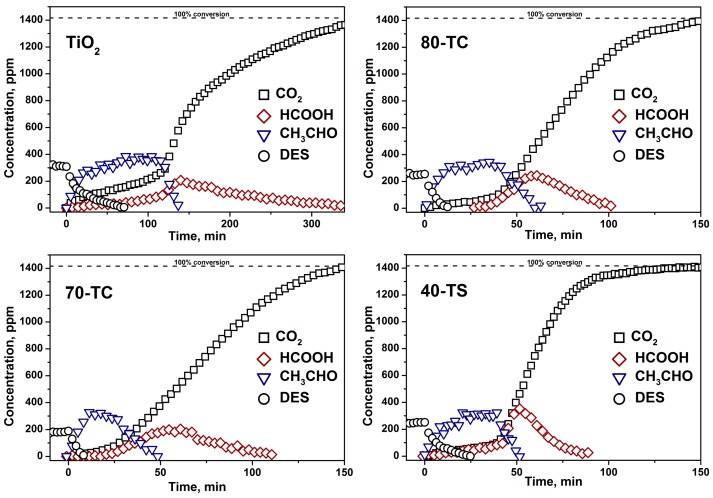
Kinetics of 0.5 μL DES PCO in the static reactor during the first cycle over the pure TiO_2_, 80-TC, 70-TC and 40-TS samples.

**Figure 7:**
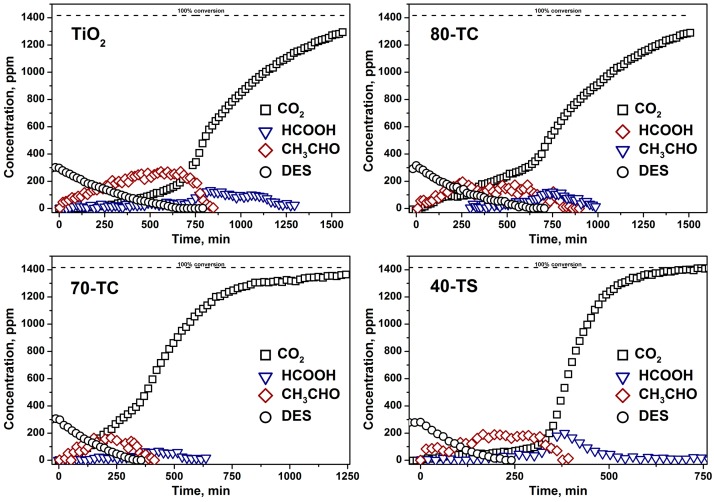
Kinetics of 0.5 μL DES PCO in the static reactor during the third cycle over the pure TiO_2_, 80-TC, 70-TC and 40-TS samples.

Kinetic curves of DES, acetaldehyde, formic acid and CO_2_ during the first and the third cycle of 0.5 μL DES PCO in the static reactor over TiO_2_, 80-TC, 70-TC and 40-TS samples are presented in the Figure 6 and Figure 7, respectively.

Besides irreversible photocatalyst deactivation caused by attachment of sulfate ions to the photocatalyst surface, temporal deactivation was also observed. In the kinetic curves this is well illustrated by the intense accumulation of acetaldehyde in the gas phase and the increase of induction period for the CO_2_ kinetic curves in the beginning of PCO run (compare the same samples in Figure 6 and Figure 7).

The reason for this temporal deactivation is the formation of partial oxidation products like diethyl sulfoxide, diethyl sulfone and others [14,16]. These non-volatile compounds accumulate on the photocatalyst surface and hinder the PCO process. The continuous photocatalyst irradiation results in the gradual oxidation of surface non-volatile species making the catalyst surface available for further DES destruction. At this moment the fast removal of acetaldehyde from the gas phase and intensive accumulation of CO_2_ begin. Formation of formic acid in the gas phase during DES PCO could be explained by its low adsorption on the photocatalyst surface due to its low molecular weight.

After the long-term irradiation complete oxidation of all intermediates was observed and the final CO_2_ concentration reached the expected 1416 ppm value calculated from the mass balance.

The use of the composite photocatalyst increases the available surface. As a result the DES removal rate and kinetics of photooxidation change. A decrease of the time needed for complete removal of DES vapor from the gas phase was observed for the composite photocatalysts. For example, in the first oxidation cycle the time values of DES removal were 73, 14, 9 and 22 min for TiO_2_, 80-TC, 70-TC and 40-TS samples, respectively. The increase of the rate of DES removal can be explained by reversible transfer of non-volatile intermediates from the TiO_2_ surface onto the support surface (AC or SiO_2_). As a result active sites on the TiO_2_ surface remained free for further interaction with DES molecules. This effect was discussed in our previous work [20].

The fast DES removal in the case of composite photocatalysts led to a decrease of the induction period of CO_2_ accumulation by about 2-fold (Figure 6). The initial rate of CO_2_ accumulation after the induction period in the first oxidation cycle was 18.9, 18.7, 14.7 and 33.2 ppm/min for TiO_2_, 80-TC, 70-TC and 40-TS samples, respectively. In contrast to the composite photocatalyst the CO_2_ accumulation rate over pure TiO_2_ sample was declining strongly with the increase of reaction time. As in the case of acetone PCO, the 40-TS demonstrated the highest activity in the DES PCO.

**Figure 8:**
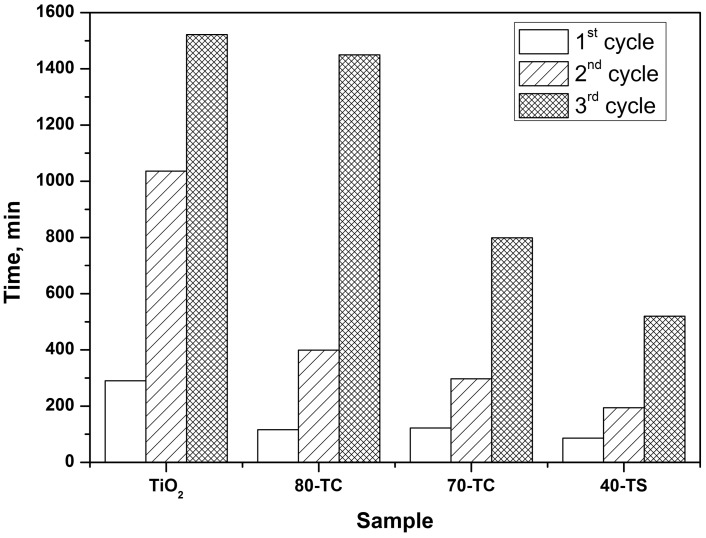
Time values of 90% DES conversion into CO_2_ in three oxidation cycles for pure TiO_2_ and TiO_2_/adsorbent composite photocatalysts.

In each subsequent oxidation cycle over the same sample a decrease of DES PCO rate was observed. As a result in the third cycle the times of complete DES removal were 600, 550, 315 and 220 min, respectively, for the pure TiO_2_, 80-TC, 70-TC and 40-TS samples (Figure 7). This means that a strong deactivation of the samples occurs. To estimate the extent of photocatalyst deactivation the time of 90% DES mineralization was calculated in each cycle. Calculated times for all samples in each oxidation cycle are presented in Figure 8.

As it follows from Figure 8, the time of the complete mineralization of DES becomes higher for each subsequent PCO cycle. For example, for the pure TiO_2_ sample in the first cycle the reaction was complete after 290 min, but in the third cycle it took 1522 min. Photocatalyst deactivation is decreasing in the following sequence: TiO_2_ > 80-TC > 70-TC > 40-TS. For example, the sum of mineralization times in all three cycles for the pure TiO_2_ sample is equal to 2848 min but for the most active and stable composite 40-TS photocatalyst it is only 800 min.

The excellent behavior of the 40-TS sample in the DES PCO can be explained by its high photocatalytic activity and large surface area which is available for the adsorption of intermediates. TiO_2_/AC samples are less active than the 40-TS sample, but are still better than pure TiO_2_ samples. It should be noted that the 70-TC sample demonstrated lower deactivation than the 80-TC sample due to its higher content of AC. In addition, the lowest concentration of gaseous intermediates, acetaldehyde and formic acid among all synthesized samples, was also observed for the 70-TC sample.

Decrease of deactivation in the case of composite photocatalyst as well as the increase of DES removal rates can be explained by reversible transfer of non-volatile intermediates, which are the reason of deactivation, from TiO_2_ particles onto the support [20]. Another possible explanation for the increased activity of the composite photocatalysts is the possible transfer of OH radicals from TiO_2_ onto the support surface. Such a possibility was shown by Carretero-Genevrier *et al*. [36], who demonstrated that OH radicals could migrate up to 10 nm distance from TiO_2_ surface into the SiO_2_ matrix. 

Finally, turnover frequency (TOF) was calculated for all samples. The total amount of mineralized DES for three consecutive runs was 3 × 0.5 = 1.5 μL or 8 × 10^18^ molecules. For all samples the amount of active component (*i.e.*, TiO_2_) was the same—3.5 mg. To estimate the surface active sites concentration we used the value of 5 × 10^14^ a.s./cm^2^ proposed by Ollis in 1980 [37].

Based on these data it is possible to calculate the total number of active sites:

(1) $$5\;\times \;10^{14}\frac{a.s.}{10^{-4}m^{2}}\times 208\frac{m^{2}}{g}\times 0.0035g=3.6\times 10^{18}\;a.s.$$

The TiO_2_ specific surface area from Table 1 was used for estimation of the active surface area because these experiments were performed at low sample quantity and we supposed that the entire photocatalyst surface was irradiated. The total time of complete DES mineralization was calculated as the sum of mineralization times in three consecutive PCO cycles presented in the Figure 8.

The estimated TON values are 1.3 × 10^−5^, 1.9 × 10^−5^, 3.0 × 10^−5^ and 4.6 × 10^−5^ s^−1^ for TiO_2_, 80-TC, 70-TC and 40-TS samples, respectively. The TOF value for 40-TS composite photocatalyst is 3.5 times higher than for pure TiO_2_ sample. Fast purification of air from the DES vapor over composite TiO_2_/SiO_2_ photocatalyst and its low deactivation during the long-term oxidation can thus be used for the development of purification methods against S-containing CWAs.

## 3. Experimental Section

### 3.1. Materials 

The following chemical reagents were used for the catalyst preparation and oxidation experiments: titanyl sulfate (TiOSO_4_∙2H_2_O, >98%, Vekton, St. Petersburg, Russia), sulfuric acid (H_2_SO_4_, 93.5%–95.6%, PKF Ant, Russia), acetone (CH_3_COCH_3_, >99.8%, Mosreaktiv, Moscow, Russia), DES (C_2_H_5_SC_2_H_5_, >98%, Fluka, Buchs, Switzerland). The reagents were used as supplied without further purification.

Activated carbon (AC) obtained by steam-gas activation of wood matter with S_BET_ = 825 m^2^/g and silica with S_BET_ = 440 m^2^/g and particle size of 10–40 μm were used for immobilization of TiO_2_ particles. AC powder (Sorbent, Perm, Russia) was boiled before synthesis in distilled water during several hours to remove ionic impurities and finally washed out thoroughly by deionized water. Silica powder was supplied from Sigma-Aldrich (St. Louis, MO, USA) and used without any treatments. Titanyl sulfate water solution with a concentration of approximately 10 wt. % was used for TiO_2_ deposition by the thermal hydrolysis method.

### 3.2. Synthesis of the Composite Photocatalyst

Composite photocatalysts were synthesized by thermal hydrolysis of TiOSO_4_ according to the procedure described in details previously [22]. Typically, a certain amount of activated carbon or silica powder was suspended in a titanyl sulfate water solution (300 mL) and boiled for 5 hours under constant mixing. The calculated TiO_2_ content in the sample was varied in the range of 65–80 wt. % for AC-containing samples and 10–80 wt. % for SiO_2_-containing samples. The TiO_2_ content was adjusted by adding a certain amount of support to 300 mL of TiOSO_4_ solution. The samples containing AC or silica were marked as X-TC or X-TS correspondingly, where X was the TiO_2_ content (wt. %). The reference TiO_2_ sample was synthesized by the same procedure without addition of support (AC or silica) and marked as TiO_2_.

### 3.3. Characterization Method 

The Ti content in the synthesized samples was determined by atomic emission spectroscopy using an Optima 4300 DV spectrometer (PerkinElmer, Waltham, MA, USA). Content of TiO_2_ was recalculated using these results according to the stoichiometric formula of oxides. The surface area and pore volume of the samples were measured by nitrogen adsorption at 77 K using the ASAP 2020 instrument (Micromeritics, Norcross, GA, USA). The specific surface area was calculated using the BET analysis and the pore volume was determined as total pore volume at P/P_0_~1. X-ray diffraction was applied to determine crystal phase composition and size of crystalline particles. XRD patterns were recorded using a D8 Advance (Bruker AXS GmbH, Karlsruhe, Germany) diffractometer with CuK_α_ radiation. The calculation of coherent-scattering domains size was performed using the Scherrer equation:

(2) $$<D>=\frac{K\lambda }{\Delta (2\theta )cos\theta }$$

with K equaled to 1.

The surface of samples and initial supports were investigated by FT-IR analysis using attenuated total reflectance technique. IR spectra of sample surface were registered using a Varian 640-IR FT-IR spectrometer (Varian Inc., Palo Alto, CA, USA) equipped by the ATR attachment. Samples were not treated in any way before analysis.

### 3.4. Kinetic Experiments

#### 3.4.1. Acetone Oxidation

Acetone oxidation was investigated in the continuous flow unit described in details previously [38]. The continuous flow unit was equipped by an IR long-path gas cell (Infrared Analysis Inc., Anaheim, CA, USA) installed in a FT801 FT-IR spectrometer (Simex, Novosibirsk, Russia). Standard operational parameters were the following: acetone concentration—20 ± 4 μmol/L, temperature—40 °C, relative humidity—22% ± 2%, volumetric flow rate (U)—0.058 L/min. Detailed information about the effect of acetone concentration on the oxidation rate in the continuous flow unit is presented in Figure S1 in the Supplementary Information.

A certain amount of sample was uniformly deposited on a glass support of 9.1 cm^2^ surface area and irradiated with UV light produced by a UV LED (Nichia, Tokushima, Japan) with λ_max_ ~373 nm. The sample irradiance in the 320–400 nm region was 9.7 mW/cm^2^. The measurement of light intensity was performed using a Spectrilight spectroradiometer (International Light Technologies, Peabody, MA, USA). The emission spectrum of the UV LED is presented in Figure S2 in the Supplementary Information section.

The concentration of acetone and CO_2_ in the reaction mixture was calculated from the FT-IR spectra using the integral form of the Beer-Lambert law:

(3) $$\int_{\omega 1}^{\omega 2}A(\omega )d\omega =\varepsilon \times l\times C$$

where $A(\omega )=\mathrm{lg}(\frac{I_{0}(\omega )}{I(\omega )})$—absorbance, *ω*_1_ and *ω*_2_—limit of the corresponding absorption band (cm^−1^), *ε* —coefficient of extinction (L·μmol^−1^·cm^−2^), *l*—optical path length (cm), *C*—gas phase concentration (μmol/L).

The rate of CO_2_ formation was used to evaluate photocatalytic activity and was calculated according to the following formula:

(4) $$W_{CO_{2}}=\Delta \;C_{CO_{2}}\times U$$

where Δ $C_{CO_{2}}$ is the difference in CO_2_ concentrations in the outlet and inlet air streams of the reactor and U is the volumetric flow rate.

#### 3.4.2. DES Oxidation

Oxidation of the DES vapor was investigated in a 0.3 L static reactor installed in the cell compartment of a Nicolet 380 FT-IR spectrometer (Thermo Fisher Scientific Inc., Waltham, MA, USA). A detailed description of the experimental setup was presented in our previous work [22].

The sample was uniformly deposited onto a 7.0 cm^2^ glass support which was placed in the reactor and irradiated with UV light produced by the UV LED described above during several hours in order to completely oxidize all organic species previously adsorbed on the catalyst surface during its storage. The sample irradiance in the 320–400 nm region was 10.2 mW/cm^2^.

After sample training 0.5 μL of liquid DES was injected into the reactor and evaporated during 30 min to achieve adsorption-desorption equilibrium. Then the UV LED was turned on and IR spectra were taken periodically. Concentrations of DES and other oxidation products in the gas phase were calculated using the Beer-Lambert law described above. The details of the quantitative calculations using IR spectra can be found in [39]. The IR spectra of individual substances which were detected in the gas phase during the DES PCO and other information which was used for calculation of extinction coefficients for each substance are presented in the Supplementary Information section in Figure S3 and Table S1.

After complete mineralization of DES in the reactor (*i.e.*, when the amount of accumulated CO_2_ reached the expected level calculated from the stoichiometric equations) the reactor was swept with fresh air and the next DES oxidation cycle was performed. Three oxidation cycles of the same amount of DES were performed for each sample.

## 4. Conclusions 

Composite TiO_2_/adsorbent photocatalysts were synthesized by the TiOSO_4_ thermal hydrolysis method in the presence of activated carbon or SiO_2_ and were tested in the photocatalytic oxidation of acetone and DES vapor. The following conclusions were made:

(1) The usage of composite photocatalyst results in up to an 8-fold decrease of DES removal time if compared with pure unmodified TiO_2_. This could be explained by an increase of the available surface area in the case of composite photocatalyst and reversible transfer of non-volatile intermediates from TiO_2_ surface to the support surface thus keeping the photocatalyst surface available for further interaction with substrate. Additionally the removal of intermediates—acetaldehyde and formic acid—occurs faster over composite photocatalyst;
(2) The long-term oxidation of DES leads to a strong deactivation of the photocatalyst. The deactivation decreases in the following sequence: TiO_2_>TiO_2_/AC>TiO_2_/SiO_2_. The most active and stable catalyst is the TiO_2_/SiO_2_ one which contains 40 wt. % of TiO_2_. The calculated TOF number for this sample is 3.5 times higher than for pure TiO_2_.

## Supplementary Materials

Supplementary materials can be accessed at: http://www.mdpi.com/1420-3049/19/12/21424/s1.
