# Supplementary material for: Photocatalytic Oxidation of Diethyl Sulfide Vapor over TiO2-Based Composite Photocatalysts
Source: Molecules. 2014 Dec 19;19(12):21424–41. doi: 10.3390/molecules191221424 (PMC6270704; doi:10.3390/molecules191221424)
Supplement: Supplementary file 1 [file molecules-19-21424-s001.pdf]

Supporting Information

1. Effect of the Acetone Concentration on the Oxidation Rate in the Continuous Flow Reactor

Experiments in the continuous flow reactor were conducted in order to investigate the dependence of acetone vapor PCO rate on the sample quantity, light intensity, substrate concentration and other parameters. Typical dependence of the acetone oxidation rate on its concentration is presented in Figure S1. After a 15 μmol/L acetone concentration further increases do not influence the PCO rate. This concentration region was chosen for investigation of synthesized samples.

Figure S1. Dependence of CO_2 formation rate during acetone oxidation in the continuous flow reactor on its concentration.

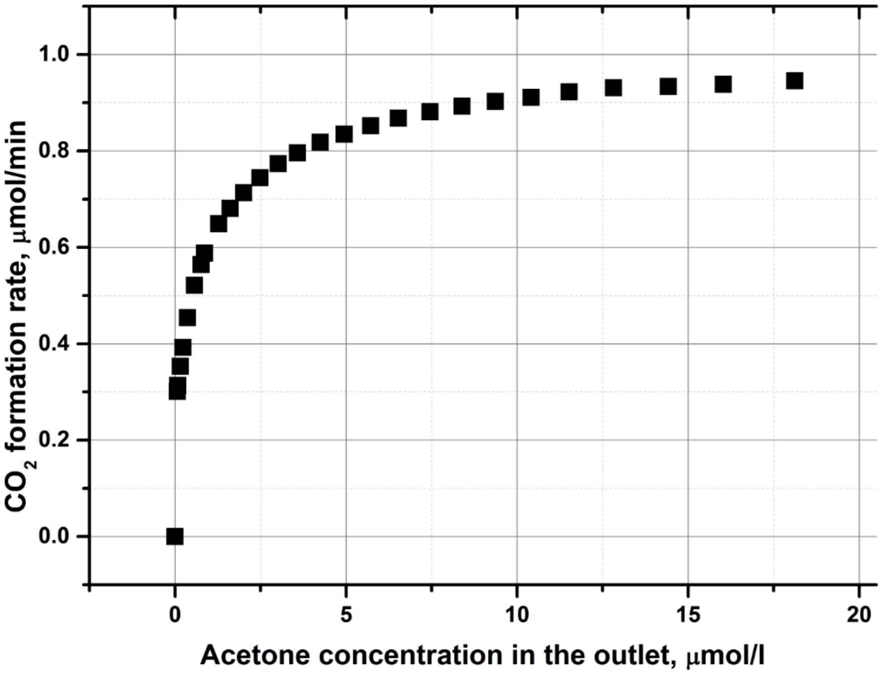

2. Emission Spectrum of the UV LED

Figure S2 presents the emission spectra of the UV LED (Nichia, Tokushima, Japan) used for the kinetic experiments in a flow circulation and in the static reactor. UVA light in the 320–400 nm range is the major part of the emission spectra of the UV LED.

Figure S2. The emission spectrum of the Nichia UV LED used for kinetic experiments. Irradiance measurement was performed at the sample location in the flow circulation reactor.

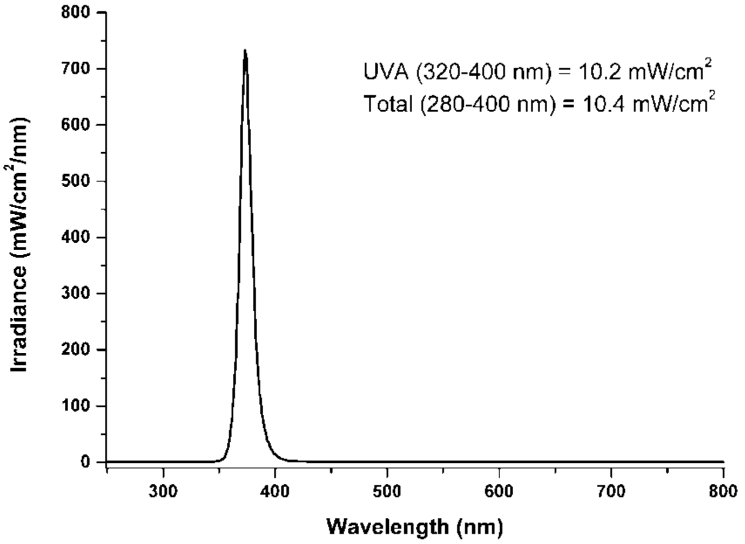

3. IR Spectra of Individual Substances which Were Detected in the Gas Phase during DES Photocatalytic Oxidation

Figure S3 shows the IR spectra of individual substances which were detected in the gas phase during DES oxidation.

Figure 3. IR spectra of individual substances in the gas phase.

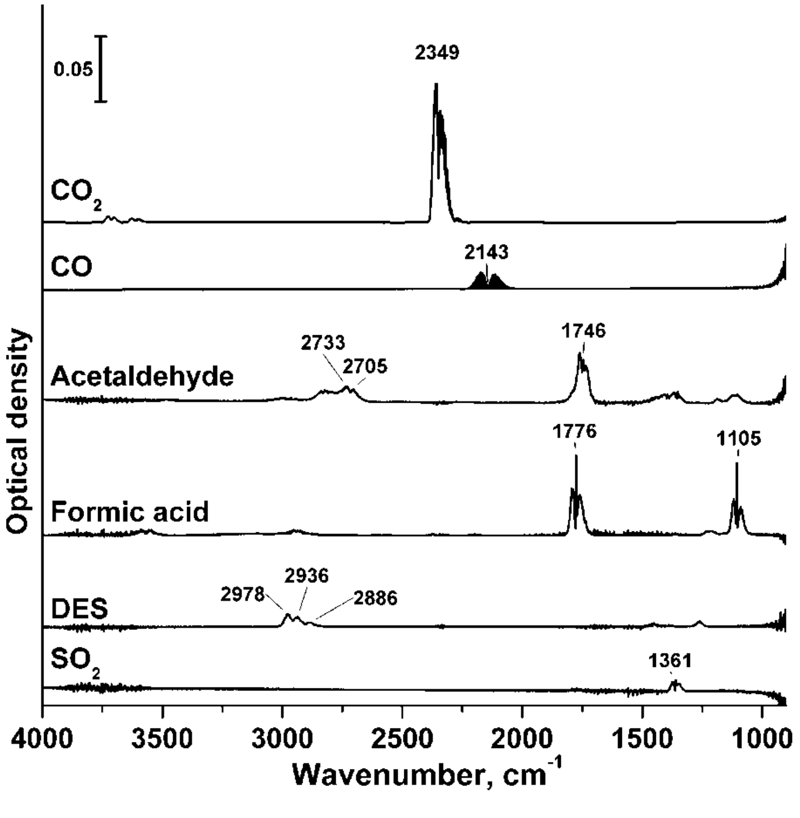

The limits of characteristic absorption bands used for calculation of concentration for each substance are presented in Table S1.

Table S1. Absorption limits and calculated coefficients of extinction.

Substance	Limits of Absorption Band, cm^{-1}		Calibration Coefficient *,	Calculated Coefficient of Extinction
	ω_1	ω_2	ppm/cm^{-1}	ε, $ppm^{-1} \cdot cm^{-2}$
Acetone	1160	1265	671	1.5×10^{-4}
Acetaldehyde	1600	1900	583	$1.7 \cdot 10^{-4}$
Diethyl sulfide	2750	3100	492	$2.0 \cdot 10^{-4}$
Formic acid	1050	1150	1913	0.5×10^{-4}
CO	2020	2250	985	1.0×10^{-4}
CO_2	2250	2450	263	3.8×10^{-4}

* The coefficient corresponds to the area of absorption band to the substance concentration obtained by the linear approximation of the calibration curve.

The details of the quantitative calculations using IR spectra can be found in [39].
